# Enhancing Knowledge Graph Extraction and Validation From Scholarly Publications Using Bibliographic Metadata

**DOI:** 10.3389/frma.2021.694307

**Published:** 2021-05-28

**Authors:** Houcemeddine Turki, Mohamed Ali Hadj Taieb, Mohamed Ben Aouicha, Grischa Fraumann, Christian Hauschke, Lambert Heller

**Affiliations:** ^1^ Faculty of Medicine of Sfax, University of Sfax, Sfax, Tunisia; ^2^ Data Engineering and Semantics Research Unit, Faculty of Sciences of Sfax, University of Sfax, Sfax, Tunisia; ^3^ Department of Communication, University of Copenhagen, Copenhagen, Denmark; ^4^ Open Science Lab, TIB-Leibniz Information Centre for Science and Technology, Hannover, Germany

**Keywords:** data mining and knowledge discovery, information retrieval and extraction, knowledge graph (ontologies), bibliometric-enhanced information retrieval, bibliographic metadata

## Introduction

Fully structured semantic resources representing facts in the form of triples (i.e., knowledge graphs) have a major function in driving computer applications, particularly the ones related to biomedicine, to library and information science and to digital humanities (Haslhofer et al., 2018; Sargsyan et al., 2020). They can be easily processed using Application Programming Interfaces (APIs, like REST APIs) and query languages (mainly SPARQL) to assess the reference semantic information and to generate accurate and precise interpretations and predictions, particularly when the analyzed data is multifactorial and ever-changing such as the COVID-19 knowledge (Turki et al., 2021c), information about the laureates of Nobel Prize in Literature (Lebuda and Karwowski, 2016), and the findings of scholarly publications (Fathalla et al., 2017). In particular, the role of open knowledge graphs to facilitate scientific collaboration has been stressed against the backdrop of the COVID-19 pandemic (Anteghini et al., 2020; Colavizza et al., 2021; Turki et al., 2021a). Effectively, the information included in textual or semi-structured resources such as electronic health records, scholarly publications, encyclopedic entries, and citation indexes can be converted into fully structured Research Description Framework (RDF) triples and included in knowledge graphs and then processed in near real-time using computer methods to obtain evolving research outputs that are automatically updated as the knowledge graphs feeding them is regularly curated. These living research outputs include systematic reviews (Wang and Lo, 2021), clinical trials (Servant et al., 2014), scientometric studies (Nielsen et al., 2017), and epidemiological studies (Turki et al., 2021b).

However, the construction of knowledge graphs is a complex effort including the recognition of scholarly publications related to the scope of the semantic resource (Turki, 2018), the retrieval of abbreviations and terms for every concept (Turki et al., 2021a), and the extraction and validation of semantic relations (Turki et al., 2018a). Many projects depend on advanced neural network-driven machine-learning techniques for applying these tasks as these methods contribute to higher quality (Asada et al., 2021; Fei et al., 2021). However, these techniques are considered as black boxes and cannot be debugged to identify the reasons behind returned false results and consequently to solve these limitations in a transparent way (Turki et al., 2021b). What is more, the quality of these techniques is considered imperfect in some cases, requiring more time to achieve the same results as specific well-defined algorithms (Turki et al., 2021b). Here, Bibliometric-Enhanced Information Retrieval (BIR) has evolved as a novel field that utilizes bibliographic metadata to efficiently drive the extraction and refinement of semantic data from scholarly publications (Cabanac et al., 2018). This field contributed to the development of many intuitive and explainable algorithms for knowledge engineering. On the one hand, this has been achieved through the restriction of the analysis of full texts to the publications including a particular value of a metadata to reveal the bibliographic settings where assessed algorithms perform well or bad (Safder and Hassan, 2019). On the other hand, this could be done thanks to the analysis of the bibliographic information using taxonomies like MeSH and Wikipedia Category Graph (Hadj Taieb et al., 2020) or using the probabilistic heuristics and constraints inferred from publications using statistical models including TF*IDF (Ramos, 2003) or extracted from knowledge graphs using inference engines, particularly HySpirit (Fuhr and Rölleke, 1998) and F-OWL (Zou et al., 2004).

In this opinion article, we explain how each type of bibliographic metadata can provide useful insights to enhance the automatic enrichment and fact-checking of knowledge graphs from scholarly publications based on the outcomes of research efforts about BIR.

## Bibliographic Metadata for Enhancing Information Retrieval From Publications

In the following subsections, we provide an overview on bibliographic metadata that plays a pivotal role when employing BIR. We selected these types of metadata based on its frequent use in BIR and its wide availability. Title, abstract, controlled keywords, citation analysis, section title as well as further metadata of scholarly publications can be easily retrieved compared to full texts that are sometimes hidden behind paywalls. As such, these types provide the opportunity for better precision and recall for information retrieval.

### Title and Abstract

To start, title and abstract are two metadata elements that can enrich knowledge graphs. Even though titles and abstracts of scholarly publications are written in natural languages and are not semi-structured as the other types of bibliographic information, they give insights about the purpose and outcomes of research outputs in a concise way. That is why they can efficiently represent the topics and the format of research papers (Stotesbury, 2003; Letchford et al., 2015). Consequently, the time-consuming and demanding natural language processing of full texts is not required when a brief analysis of titles and abstracts can return required information for information retrieval purposes. As such, the availability of open abstracts as research data has been recently emphasized by the Initiative for Open Abstracts (I4OA) (Tay et al., 2020).

The application of feature-based measures of sentences’ semantic similarity to compare the titles or abstracts of two scholarly publications can be efficient to identify whether the two papers describe similar topics or not (Hadj Taieb et al., 2019; Hadj Taieb et al., 2020) and this can serve to contextualize the co-citation and citation links between papers as well as to filter term co-occurrences for a more precise knowledge graph construction and refinement (Hadj Taieb et al., 2020). This is particularly true for the domain of STEM (Science, Technology, Engineering, and Mathematics) since it features, compared to arts and humanities as well as social sciences, an agreed-upon vocabulary which mostly addresses directly its subjects. Semantic similarity measures are driven by external knowledge resources like knowledge graphs and ontologies and can consequently compare brief texts with high accuracy and speed (Hadj Taieb et al., 2015) and full transparency (Turki et al., 2021b) by contrast to other advanced techniques applied to full texts, particularly deep learning, semantic embeddings (Sargsyan et al., 2020), TF-IDF^1^ (White, 2018), and Latent Dirichlet Allocation (Jeong et al., 2014).

The consideration of the format of the titles and abstracts when applying information retrieval techniques can be an important factor for advancing the state of the art of the knowledge engineering field. The letter case of words in titles and abstracts can be useful for many information retrieval applications. For example, it can help identify scholarly abbreviations that are generally written in uppercase letters (Zhou et al., 2006) or to extract structured abstracts including uppercase section titles (Ripple et al., 2011). Such algorithms should be considered with care, particularly when the title or the abstract is fully written in uppercase letters such as in Telford et al. (1985). In this situation, case-sensitive algorithms should not be applied to uppercase titles and abstracts as this can alter the efficiency of the methods. Such systems cannot even be applied to the full text of a scholarly publication when both the title and the abstract are in uppercase letters. Consequently, such decisions can only be made by human reading of the title, abstract, and full text, if necessary.

The restriction of several natural language processing algorithms to the titles and abstracts of scholarly publications can be associated with higher accuracy rates for methods. The usage of several patterns in titles and abstracts, particularly parentheses, can be less complicated in titles and abstracts than in full texts and this explains in part the higher accuracy of parenthetic abbreviation extraction from titles (Zhou et al., 2006). For instance, there are more situations where parentheses are used in full texts for explaining facts, mentioning in-text references and defining *p*-values for evaluating assumptions when parentheses are mainly used in titles for stating abbreviations and declaring chemical formulas (Zhou et al., 2006). This phenomenon should raise concerns about the application of information retrieval methods only tested on titles and abstracts to full texts on the one hand and detailed guidelines for deciding when the used methods should be restricted to titles and abstracts to obtain a better precision and recall for information retrieval.

### Controlled Keywords, Citation Analysis, and Section Title

As a rule, richer metadata on publications are available than presented in the previous section. This includes contextual information such as content classifications, relationships to other documents as well as structure of content within an article.

Regarding classification of the publication as a whole, controlled keywords are featured as terms from a reference terminology that are used to label scholarly publications in several bibliographic databases. Examples of these keywords are KeywordPlus attributed to Web of Science records (Zhang et al., 2016) and MeSH Keywords assigned to PubMed publications (Turki et al., 2018a). The advantage of using these keywords is that they allow the use of a unique term and not of synonyms to assign each concept to publications and allow to prevent the redundancy of many variants of the same term across the maintained citation index allowing a more precise data mining and knowledge engineering of the bibliographic database (Henry and McInnes 2017; Turki et al., 2018a). That is why the co-occurrence analysis of controlled keywords, particularly MeSH Keywords, is nowadays used in semantic relation extraction and validation and provides a high accuracy rate for such an action (Henry and McInnes, 2017). Concerning MeSH Keywords, the recognition of a semantic relation can be achieved through the identification of the compatibility of the qualifiers of two significantly co-occurring keywords (e.g., Sofosbuvir/*therapeutic use* and Hepatitis C/*drug therapy*), of the complementarity of the qualifier of a keyword with the class of the heading of another largely co-occurring keyword [e.g., Sofosbuvir/*therapeutic use* and Hepatitis C (*disease*)], or of the association of the classes of the headings of two mainly linked keywords [e.g., Sofosbuvir [*drug*] and Hepatitis C (*disease*)] (Turki et al., 2018a).

Despite the fact that citations also have some shortcomings, they are currently recognized as major information in Scientometrics as they can provide important details about the impact of scholarly publications as well as the evolution of scientific outcomes (Zhai et al., 2018). That is why they can be useful for refining and enriching the outputs of information retrieval from research papers. As the majority of research publications is most likely to be cited by and co-cited with related papers dealing with the same topic, initially considered papers for constructing and validating a knowledge graph can be odd ones and should not be processed if they do not belong to the citation or co-citation network of the topic of the semantic resource (Turki, 2018). By contrast, the papers that have the best centrality in the citation or co-citation network of the field of the knowledge graph should be considered as reference resources that should drive the beginning and reasoning of the information retrieval algorithms as these publications are the main papers in the field upon which all other papers have been developed (Diallo et al., 2016).

Controlled keywords and citations can be combined together to provide an added value to knowledge graph creation and validation from scholarly publications. The sentence including an inline citation to a work can be a key for enriching information about the citing paper as well as the cited one (Aljaber et al., 2011). The controlled keywords and the title analysis of the cited paper can be used to enrich the semantics of the citing sentences and recognize a hidden scientific relation or entity that has been discussed without being clearly stated (Aljaber et al., 2011; Hadj Taieb et al., 2020). The analysis of the inline citation using automatic named entity annotation and scientific relation embedding can reveal controlled vocabularies and relations that are not originally used to describe the cited paper in bibliographic databases (Aljaber et al., 2011).

In another context, several types of semantic relations are available in particular sections of a scholarly publication (Turki et al., 2018a; Alexander and de Vries, 2021). For example, information about research funding for a given paper can only be found in specific parts (Alexander and de Vries, 2021). Alexander and de Vries (2021) note that the choice of an algorithm is important at the beginning of a research project. They use their algorithm to extract funding information from scholarly publications. The advantage of this text is that it is typically included under the section “Acknowledgments” or “Funding Information” in scholarly publications and adheres to certain writing standards, for instance, “This research is funded by’” followed by the name of the research funder, in some cases the funding program, and finally the grant number.

Similarly, the section titles in narrative literature reviews provide an outlook about the information included in each part where a section entitled “Symptoms” in a review about Hepatitis C includes semantic relations about the symptoms of the described medical condition (Turki et al., 2018a). Subsequently, considering section titles during semantic relation extraction and validation can not only reduce the complexity of the recognition of the relation types but also minimize the time allocated for such a task by restricting the process to the sections that are expected to include the required relations.

### Other Metadata

What other metadata elements of scholarly publications can be considered when it comes to building knowledge graphs? Scholarly publications have different levels of evidence according to their settings, the age, the type, the status, the research area, and the source title of a given output. All these factors may influence the significance of its findings to the research community (Burns et al., 2011). The age of a research paper can typically determine whether the information included in it is outdated or not as terms and abbreviations might change over years due to nomenclature updates (AlRyalat et al., 2018) and as several findings can be disproven after a time period thanks to advances in experimentation techniques and scientific reasoning (Arbesman, 2013). Although science is an ever-evolving enterprise, it is also based on certain classical literature, for example, the importance of the founders of academic disciplines, such as sociology. Consequently, scientists have to be mainly based on new publications to create better and updated knowledge graphs about their topics of interest and even to predict the evolution of the constructed knowledge graph in the next years (Choudhury et al., 2020). The type of a given publication can affect the amount and quality of information it includes. When letters only present a limited number of facts in a few pages (Turki et al., 2018b), reviews provide a detailed overview of the concepts and findings related to a given topic from the synthesis of many papers and are consequently more adequate as resources for scholarly information retrieval (Burns et al., 2011; Turki et al., 2018a).

Acronyms used in scholarly literature can serve as a reliable goal of matching concepts in a research field. Many acronyms seem to be established terminology that is referred to frequently, in an unambiguous way. It has been shown that even for large corpuses of scientific papers from diverse fields automated disambiguation can be applied unsupervized and at scale (Charbonnier and Wartena 2018; Veyseh et al., 2021).

The status of a research publication can be also important for ensuring the quality of the extraction of scientific knowledge. Although bibliographic databases like *PubMed* ^2^ and features like *Crossmark* ^3^ state whether a publication is a preprint or a partially or fully retracted paper, most of the projects for the creation and validation of knowledge graphs do not consider this factor when retrieving facts from research papers (Sargsyan et al., 2020). The matter with considering preprints in information retrieval is that these publications have not undergone peer review (Glasziou et al., 2020) and their outputs can be dynamically changed over months (Oikonomidi et al., 2020). That is why using them to generate structured information about a given topic can harm the quality of the created resource and make it less trustworthy. As for retracted publications, they are papers that have been proven to include false or fabricated claims, and were rejected by the scientific community and eliminated from their journal of publication for this reason. Although retractions are continuously cited for various reasons (e.g., lacking knowledge about the retractions), applying information retrieval techniques on them can let the users of the returned semantic data reuse scientifically doubtful findings and probably make wrong interpretations (Sotudeh et al., 2020; Soltani and Patini, 2020).

The restriction of the set of considered publications according to their research areas as revealed in citation indexes or through the analysis of author keywords allows refining the creation and validation of knowledge graphs by eliminating outputs outside the scope of the developed resource (Salatino et al., 2020). This prevents the overlapping of concepts from different fields when they are represented by the same polysemous term and consequently eliminates noise from the generated database. To be precise, a polysemous term has various meanings. The consideration of the research venue of publications can be also efficient in this context. Further than the ability to analyze source titles using semantic similarity measures, among other techniques, to verify the topics of interest of journals and conferences (Hadj Taieb et al., 2015), metrics about the venues such as the journal impact factor and the number of citations can be used to filter the considered sources and only consider the most prestigious and reliable ones (Pal, 2021). While the journal impact factor has shortcomings, it can be a useful indicator in this context.

## Conclusion

In this opinion article, we showed the kinds of semantic information that can be revealed from each type of bibliographic metadata and that can be later used to strengthen information retrieval for knowledge graph construction and validation from scholarly publications. Given this, we invite the scientific community to collaborative projects considering bibliographic information when extracting domain information from scholarly publications for the creation and validation of trustworthy and precise semantic resources. As a future direction of this work, we suggest investigating how bibliographic metadata can enhance information retrieval algorithms using a series of experiments comparing the accuracy of methods processing full texts of scholarly publications with the one of bibliometric-enhanced information retrieval approaches. We consequently propose to study how bibliometric-enhanced information retrieval can enhance knowledge graph construction and validation as well as other interesting computational tasks such as predicting future scientific breakthroughs and major prize winners, natural language generation and translation of scholarly texts, and the automation of the creation and update of various kinds of research outputs. Researchers may also consider the adaptation of BIR algorithms to support the augmentation of university-level courses and evaluation quizzes with explanatory excerpts from scholarly outputs and to recommend scholarly publications to fight online misinformation. As well, we recommend building a framework for explainable artificial intelligence that returns explanations of the use of machine learning models for a given task based on what is currently available about the matter in research papers. As far as the availability of the data needed for BIR is concerned, it is to be hoped for the future that initiatives such as the I4OA mentioned above will gain momentum and that the applicability and re-usability of bibliographic metadata for BIR will become easier.

## Footnotes

1 TF-IDF: term frequency–inverse document frequency.

2 Further details can be found in the PubMed User Guide at https://pubmed.ncbi.nlm.nih.gov/help/. For PubMed, information about the status of a research publication can be automatically retrieved using Biopython (Chapman and Chang, 2000).

3
*Crossmark* is a crowdsourcing tool and database developed by CrossRef Organization that allows to annotate the types and statuses of scholarly publications (Meyer, 2011).
